# Loss and Gain of Natural Killer Cell Receptor Function in an African Hunter-Gatherer Population

**DOI:** 10.1371/journal.pgen.1005439

**Published:** 2015-08-20

**Authors:** Hugo G. Hilton, Paul J. Norman, Neda Nemat-Gorgani, Ana Goyos, Jill A. Hollenbach, Brenna M. Henn, Christopher R. Gignoux, Lisbeth A. Guethlein, Peter Parham

**Affiliations:** 1 Departments of Structural Biology and Microbiology & Immunology, Stanford University School of Medicine, Stanford, California, United States of America; 2 Department of Neurology, University of California, San Francisco, California, United States of America; 3 Department of Ecology and Evolution, Stony Brook University, Stony Brook, New York, United States of America; 4 Department of Genetics, Stanford University School of Medicine, Stanford, California, United States of America; University of Pennsylvania, UNITED STATES

## Abstract

Modulating natural killer cell functions in human immunity and reproduction are diverse interactions between the killer cell immunoglobulin-like receptors (KIR) of Natural Killer (NK) cells and HLA class I ligands on the surface of tissue cells. Dominant interactions are between KIR2DL1 and the C2 epitope of HLA-C and between KIR2DL2/3 and the C1 epitope of HLA-C. KhoeSan hunter-gatherers of Southern Africa represent the earliest population divergence known and are the most genetically diverse indigenous people, qualities reflected in their *KIR* and *HLA* genes. Of the ten KhoeSan *KIR2DL1* alleles, KIR2DL1*022 and KIR2DL1*026 likely originated in the KhoeSan, and later were transmitted at low frequency to the neighboring Zulus through gene flow. These alleles arose by point mutation from other KhoeSan *KIR2DL1* alleles that are more widespread globally. Mutation of KIR2DL1*001 gave rise to KIR2DL1*022, causing loss of C2 recognition and gain of C1 recognition. This makes KIR2DL1*022 a more avid and specific C1 receptor than any KIR2DL2/3 allotype. Mutation of KIR2DL1*012 gave rise to KIR2DL1*026, causing premature termination of translation at the end of the transmembrane domain. This makes KIR2DL1*026 a membrane-associated receptor that lacks both a cytoplasmic tail and signaling function. At higher frequencies than their parental allotypes, the combined effect of the KhoeSan-specific KIR2DL1*022 and KIR2DL1*026 is to reduce the frequency of strong inhibitory C2 receptors and increase the frequency of strong inhibitory C1 receptors. Because interaction of KIR2DL1 with C2 is associated with risk of pregnancy disorder, these functional changes are potentially advantageous. Whereas all other KhoeSan *KIR2DL1* alleles are present on a wide diversity of centromeric *KIR* haplotypes, KIR2DL1*026 is present on a single *KIR* haplotype and KIR2DL1*022 is present on two very similar haplotypes. The high linkage disequilibrium across their haplotypes is consistent with a recent emergence for these *KIR2DL1* alleles that have distinctive functions.

## Author Summary

The genes that control the response of the human immune system vary enormously between individuals. Understanding the evolution of these genetic differences and how they individualize immune responses is central to understanding how the immune system works in health and disease. In this regard, the KhoeSan of southern Africa are particularly informative because they are genetically diverse, divergent from other modern human populations and have been subject to unique demographic history. In the KhoeSan population, we studied variable genes that control natural killer cell function. We identified two recently evolved, novel gene variants that have unusual function; one completely changed its ligand specificity and the other lost its capacity for signal transduction.

## Introduction

Natural killer (NK) cells are versatile lymphocytes that contribute to reproduction and immune defense [1,2]. Modulating the activities of human NK-cells are the killer-cell immunoglobulin-like receptors (KIR). These receptors engage the HLA class I ligands (HLA-A, -B and -C) expressed on the surface of most human cells. Such interactions direct NK cells to kill virus-infected cells and tumor cells; they also induce the secretion of cytokines that activate other leukocytes or guide fetal trophoblast cells to invade the uterus during pregnancy. In human populations, both receptors and ligands are highly polymorphic. Their combinatorial diversity contributes to the resistance of individuals to infection, and their susceptibility to autoimmunity and pregnancy syndromes [1,3]. A minority of HLA-A and -B allotypes are ligands for KIR, whereas all HLA-C allotypes fulfill this role. HLA-C arose more recently than HLA-A and -B and has evolved to become the predominant polymorphic KIR ligand [4]. In reproduction it is the only polymorphic ligand, because HLA-C is expressed by fetal trophoblast cells whereas HLA-A and -B are not [1].

KIR engage the upward face of the HLA class I molecule formed by the α_1_ domain, the α_2_ domain and the bound peptide antigen [5]. αβ T cell receptors engage the same face, in an overlapping but different way [6]. Dimorphism at position 80 in the α_1_ domain of HLA-C defines two mutually exclusive epitopes, C1 (asparagine 80) and C2 (lysine 80), recognized by different KIR [7]. All the numerous (>1,700) HLA-C allotypes have either the C1 or C2 epitope. Human KIR are comprised of four phylogenetic lineages, of which the KIR that recognize HLA-C are all lineage III [4]. They have two extracellular immunoglobulin-like domains (D1 and D2), which together form the site that binds HLA-C [5]. Within the binding site, dimorphism at position 44 in the D1 domain determines if a KIR is specific for C1 (lysine 44) or C2 (methionine 44). *KIR2DL1* and *KIR2DS1* encode inhibitory and activating C2 receptors, respectively. *KIR2DL2/3* encodes inhibitory C1 receptors. (There is no activating C1 receptor). The inhibitory receptors are highly polymorphic, with 25 KIR2DL1 and 36 KIR2DL2/3 variants being defined, whereas KIR2DS1 with seven variants is relatively conserved.

Among individuals and populations, *KIR* are further diversified by gene content variation [8]. Whereas *KIR2DL2/3* is present on almost every human *KIR* haplotype described, neither *KIR2DL1* nor *KIR2DS1* are present on every haplotype. Represented in every human population are two distinctive *KIR* haplotype groups: *A* and *B* [1]. KIR *A* haplotypes encode high avidity inhibitory receptors for HLA class I and have one activating receptor gene; *B* haplotypes encode low avidity inhibitory receptors for HLA class I and have several activating receptor genes. This bipartite system of functionally distinctive *KIR* haplotypes appears unique to humans because it is not present in chimpanzees or any other species investigated [9].

For reasons of practicality, the functional properties of KIR2DL1 and KIR2DL2/3 have been studied mainly in the context of allotypic variants that combine high avidity, high specificity and high frequency in Europeans [1]. In contrast, for sub-Saharan African populations, which have the highest genetic diversity [10–12] and among the highest mortality from infectious disease and pregnancy complications [13], KIR investigation is in its infancy and has so far focused on West African and Bantu-speaking populations [14]. Within sub-Saharan Africa, some indigenous populations are as different from each other as they are from Europeans [10,12,15]. Notably, the KhoeSan who reside across southern Africa descend from the deepest human population divergence and have among the greatest genetic diversity of any population [10,16,17]. During the last 2,000 years there has been admixture between the KhoeSan and Bantu-speaking agriculturalists who expanded southwards [11,18]. More recently, the arrival of European colonists over the past 500 years has introduced novel infectious diseases including smallpox and tuberculosis [19]. Here we describe high-resolution genetic and functional studies on the HLA-C specific KIR of the KhoeSan and their comparison to other populations.

## Results

### Two unusual *KIR2DL1* alleles evolved in the KhoeSan after their divergence from other modern humans

**Fig 1:**
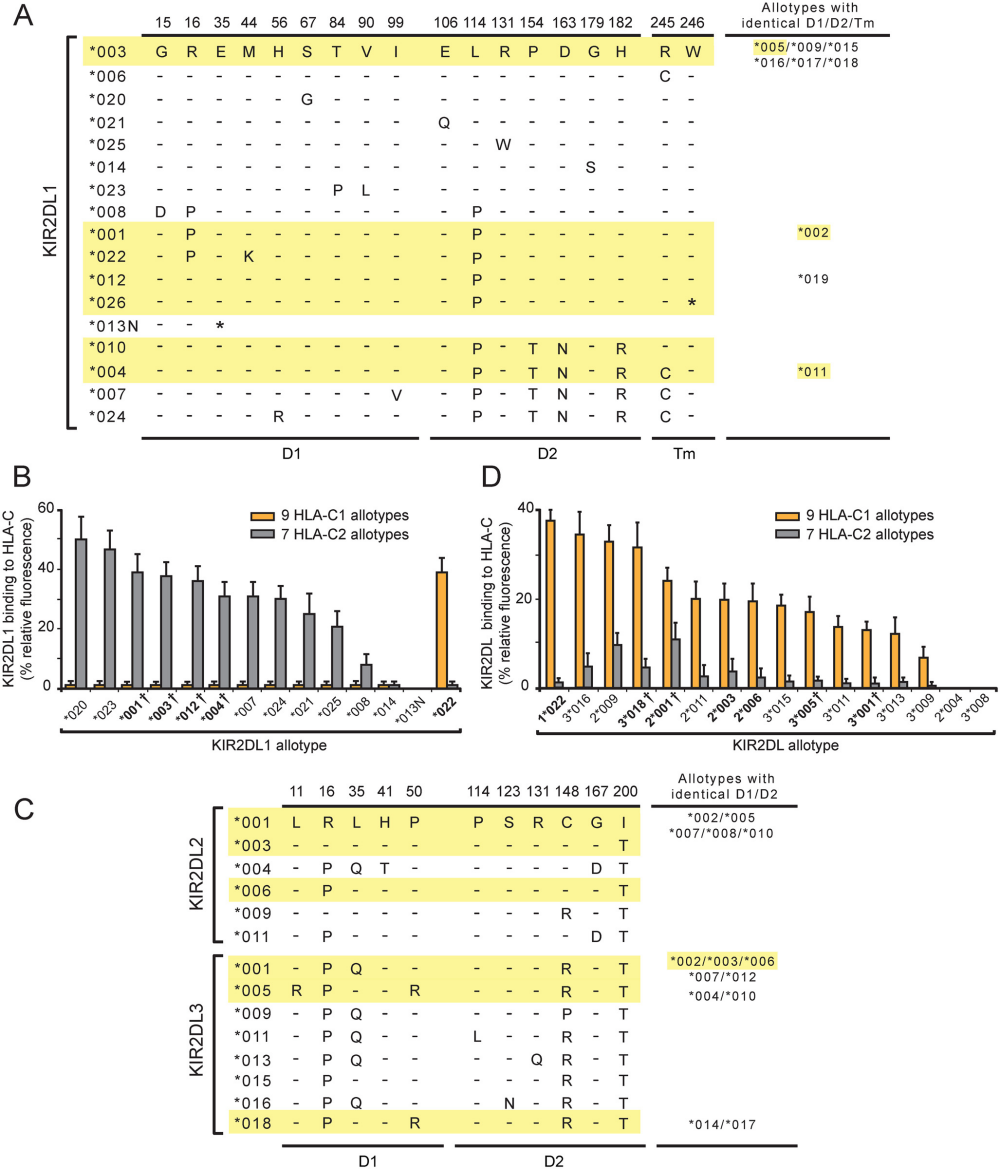
A variant of KIR2DL1 originating in the KhoeSan is a C1-specific receptor and not a C2-specific receptor like other KIR2DL1. (A) This alignment of KIR2DL1 sequence differences shows the sites of polymorphism in the D1 domain (D1), the D2 domain (D2) and the transmembrane region (Tm). Dashes denote identity with the KIR2DL1*003 sequence, an asterisk denotes a termination codon. Sequences of the KhoeSan KIR2DL1 allotypes are highlighted in yellow. The names of allotypes with D1, D2 and Tm identical to an aligned sequence are listed in the column at the right. (B) Binding of KIR2DL1-Fc fusion proteins to microbeads coated with C1-bearing and seven C2-bearing HLA-C allotypes. Each binding value was normalized to that of the W6/32 antibody and these normalized values were averaged for the C1 (N = 9) and C2 (N = 7) allotype groups. The names of allotypes present in the KhoeSan are boldened. A dagger following the listed allotype indicates that the allotype represents a group of two or more alleles that encode identical ligand binding domains (see Panel A). (C) This alignment of KIR2DL2/3 sequence differences shows the sites of polymorphism in the D1 and D2 domains. Dashes denote identity with the KIR2DL2*001 sequence Sequences of the KhoeSan KIR2DL2/3 allotypes are highlighted in yellow. The names of allotypes with D1 and D2 identical to an aligned sequence are listed in the column at the right. (D) Binding of KIR2DL2/3-Fc fusion proteins to microbeads coated with C1-bearing and C2-bearing HLA-C allotypes. Each binding value was normalized to that of the W6/32 antibody and these normalized values were averaged for the C1 (N = 9) and C2 (N = 7) groups. The names of allotypes present in the KhoeSan are boldened. Groups of allotypes with identical D1 and D2 domains, and which are represented by a single KIR2DL2/3-Fc, are as shown in the column on the right of Panel A. A dagger following the listed allotype indicates that the allotype represents a group of two or more alleles that encode identical ligand binding domains (see Panel A).

From analysis of 61 KhoeSan we identified ten *KIR2DL1* alleles (Fig 1A and S1A Fig). Of these, 2DL1*022 and 2DL1*026 are new discoveries that have frequencies in the KhoeSan of 17.2% and 4.2%, respectively. Being absent from all previously studied populations [14,20–24], suggested that 2DL1*022 and 2DL1*026 are specific to the KhoeSan. To test this hypothesis we examined additional populations for the presence of these alleles. We first examined data from the 1000 Genomes project dataset [25] by probing for sequence-specific reads that correspond to the 2DL1*022 and 2DL1*026 alleles. *KIR3DL3*, a framework gene present on all *KIR* haplotypes, served as the positive control. All 2,496 individuals sampled had reads corresponding to *KIR3DL3*, as did the eight KhoeSan who were analyzed similarly by whole-exome sequencing [26]. The eight KhoeSan individuals also gave allele-specific *KIR2DL1* reads consistent with their high-resolution *KIR2DL1* genotype (S2A Fig). In this context it is striking that none of the 2,496 individuals, representing 26 different populations worldwide (S2B Fig), was found to have either 2DL1*022 or 2DL1*026 (S2C Fig). Because the 1000 Genome dataset represents a limited subset of sub-Saharan African population diversity [12,27], we expanded our search to include four further groups.

To determine if 2DL1*022 or 2DL1*026 are present in African hunter-gatherer populations other than the KhoeSan we examined three groups: the Hadza who are an isolated click-speaking population that live in northern Tanzania [10] and the central African Mbuti and Baka Pygmies. Together with the KhoeSan, these hunter-gatherer groups may have formed a larger proto-KhoeSan-Pygmy population prior to their divergence 50,000–100,000 years ago [12,28,29]. Neither 2DL1*022 nor 2DL1*026 was detected in any Hadza or Pygmy individual (S2C Fig). Despite the relatively low number of individuals sampled (52 Hadza and 40 Pygmies) this result indicates that 2DL1*022 and 2DL1*026 are not present at any appreciable frequency in these groups.

To determine whether 2DL1*022 and 2DL1*026 are present in other southern African populations, we examined 100 Zulu individuals whose genomes were sequenced as part of the African Genome Variation Project [27]. With the same approach used to probe the 1000 Genomes dataset, we identified three Zulus having 2DL1*022 and two having 2DL1*026 (S2C Fig). As these five individuals were all *2DL1* heterozygous, we estimate that the frequencies of 2DL1*022 and 2DL1*026 in the Zulu population are approximately 1.5% and 1%, respectively. Examination of the centromeric half of the *KIR* haplotypes, the location of the *KIR2DL1* gene, showed that each Zulu allele is likely present on the identical haplotype background to that found in the KhoeSan (S3 Fig). Together with their low frequencies, this suggests that these alleles were introduced into the Zulu population as a result of admixture with KhoeSan hunter-gatherers. This interpretation is supported by studies that have demonstrated recent KhoeSan admixture with the Zulus [10,11,18,27] and by the absence of both 2DL1*022 and 2DL1*026 from a Bantu-speaking population in east Africa (Kenyan Luhya from the 1000 Genomes dataset). These data support an evolutionary model in which 2DL1*022 and 2DL1*026 rose in frequency in the KhoeSan populations sometime after their divergence from the other groups and thus within the past 100,000 years [12,28,29].

KIR2DL1*022 differs from 2DL1*001, also present in the KhoeSan, by a single non-synonymous substitution in codon 44 (Fig 1A). Thus 2DL1*022 likely evolved from 2DL1*001 by a point mutation that caused methionine to be replaced by lysine at position 44 (Fig 1A). Position 44 dimorphism determines whether a given KIR2DL has specificity for the C1 or C2 epitope of HLA-C [7]. Prior to investigation of the KhoeSan, all the known KIR2DL1 allotypes (n = 23) had methionine 44 and were predicted to be C2-specific. Conversely, and in complementary fashion, the known KIR2DL2/3 allotypes (n = 36) all had lysine 44 and were predicted to be C1 specific. In this context, 2DL1*022 appears an extraordinary KIR2DL1 allotype, being predicted to be a C1 receptor and not a C2 receptor like other KIR2DL1 allotypes. Thus, the mutation that created 2DL1*022 had two important functional effects: loss of C2 recognition and gain of C1 recognition.

KIR2DL1*026, the other KhoeSan-specific *KIR2DL1* allele, differs from 2DL1*012, also present in the KhoeSan, by one nucleotide substitution. Thus KIR2DL1*026 likely arose from 2DL1*012 by point mutation. This substitution converted the tryptophan codon at position 246 to a termination codon (Fig 1A). Position 246 is situated at the boundary between the transmembrane domain and the cytoplasmic tail. Consequently, 2DL1*026 lacks the immunoreceptor tyrosine-based inhibitory motifs of the cytoplasmic tail that mediate inhibitory signaling function [30]. Less obvious is the effect that absence of a cytoplasmic tail could have on the association of 2DL1*026 with cellular membranes. Thus, the mutation that created 2DL1*026 clearly has a major effect in abrogating inhibitory signaling function, but it also has potential to alter the amount of receptor that reaches the NK cell-surface.

### KhoeSan specific 2DL1*022 is an unusually strong and specific C1 receptor

To determine the avidity and specificity of 2DL1*022 for HLA class I, and also to compare its binding reactivity with other KIR2DL1 allotypes, we made a panel of 14 KIR2DL1-Fc fusion proteins that covers the allotypic range of KIR2DL1 binding sites (Fig 1A). Each KIR-Fc was tested for binding to a panel of 97 microbeads in which each bead is coated with one of 31 HLA-A, 50 HLA-B and 16 HLA-C allotypes. Our previous work has shown that the results obtained with this cell-free bead-binding assay correlate well with those derived from in vitro functional assays of NK cell cytotoxicity [31,32].

Assessment of the pairwise interactions between 14 KIR2DL1-Fc and 16 HLA-C allotypes shows that 2DL1*022 binds to all nine C1-bearing HLA-C allotypes but to none of the seven C2-bearing HLA-C allotypes in the test panel (Fig 1B). KIR2DL1*022 also binds HLA-B*46:01 and HLA-B*73:01, two exceptional C1-bearing HLA-B allotypes but to no other HLA-B allotype, or any HLA-A allotype. Eleven HLA-C1 bearing allotypes are present in the KhoeSan (S1C Fig). Neither HLA-B*46:01 or HLA-B*73:01 are present in the KhoeSan, their distributions being focused on Southeast Asia (B*46:01) or West Asia (B*73:01) [9,33,34]. As we predicted, 2DL1*022 functions as a C1-specific receptor and not a C2-specific receptor like eleven of the 13 other KIR2DL1-Fc. These eleven KIR2DL1-Fc molecules varied in their avidity for C2 by half an order of magnitude. In contrast, 2DL1*013N-Fc and 2DL1*014-Fc bound to no HLA class I allotype (Fig 1B). For 2DL1*013N this result was anticipated, because the protein is a fragment that terminates prematurely at residue 34 in the D1 domain. On the other hand, 2DL1*014 was expected to bind HLA class I, because it differs from 2DL1*003 only by substitution of glycine for serine at position 179 in the D2 domain (Fig 1A). Neither the 2DL1*013N nor the 2DL1*014 allotype is present in the KhoeSan. Overall, these results vividly illustrate how the natural polymorphism of KIR2DL1 modulates the avidity, specificity and functionality of this NK cell receptor in human populations.

In the KhoeSan, mutation of 2DL1*001, a strong C2 receptor, produced the C1 receptor, 2DL1*022. We therefore examined how the properties of 2DL1*022 compare to the prototypical C1 receptors encoded by the *KIR2DL2/3* gene. (This gene has two distinctive allelic lineages, *2DL2* and *2DL3*, hence the *KIR2DL2/3* name). KIR-Fc proteins were made from six 2DL2 and nine 2DL3 allotypes (Fig 1C) and their binding to HLA class I coated beads was compared to 2DL1*022 (Fig 1D). As a group, the KIR2DL2/3 allotypes are not as specific for C1 as the KIR2DL1 allotypes are for C2. KIR2DL2/3 exhibit a range of avidity for C1, but increasing avidity for C1 is accompanied by increased cross-reactivity with C2 (Fig 1D). This is, however, not the case for 2DL1*022, which has a higher avidity for C1 than any of the KIR2DL2/3 allotypes, but no significant C2 cross-reactivity. KIR2DL1*022 has completely lost recognition of C2 while gaining a stronger, more specific, recognition of C1 than any KIR2DL2/3 allotype. Thus KIR2DL1*022 is seen to have unique functional properties, ones that will clearly have a profound functional impact on the KhoeSan and Zulu individuals who carry this allele.

**Fig 2:**
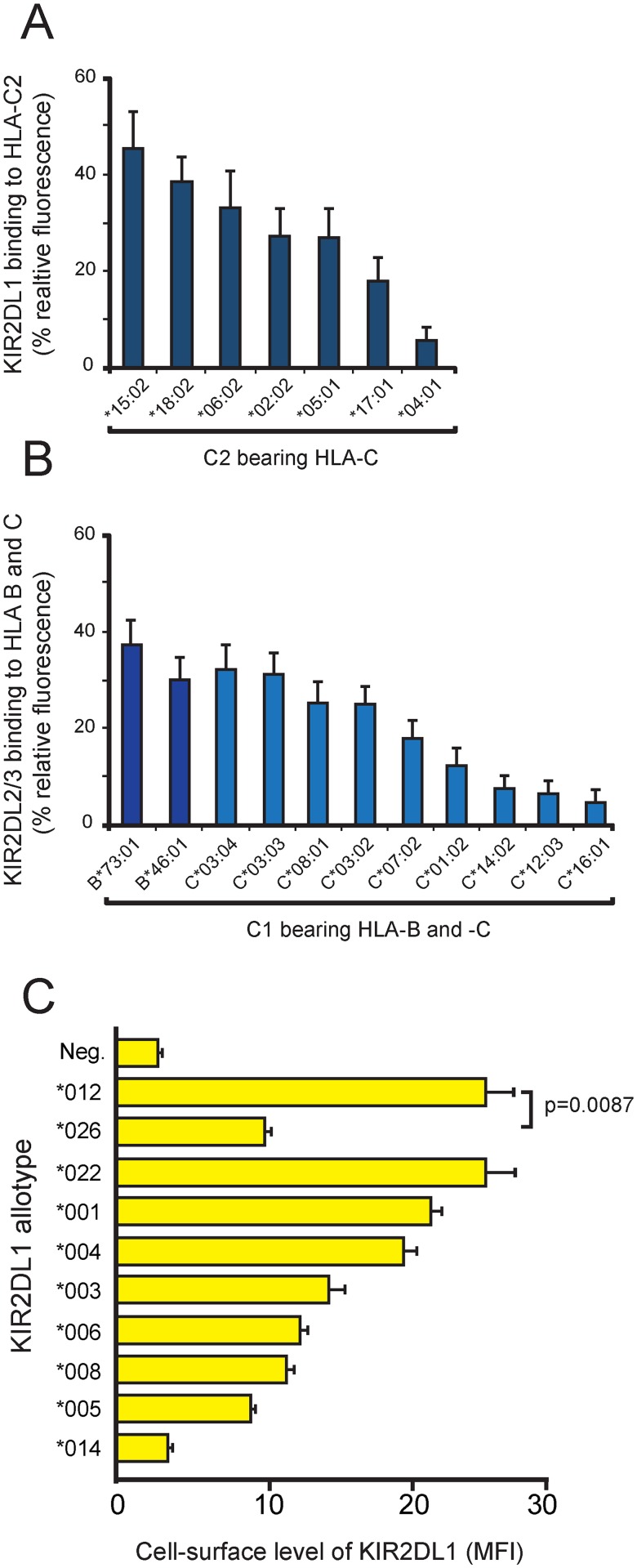
KIR polymorphism modulates the avidity and specificity for HLA-C, as well as KIR abundance at the cell surface. (A) Binding of KIR2DL1-Fc fusion proteins to C2-bearing HLA-C allotypes. For each C2-bearing HLA-C allotype, the KIR2DL1 binding is the mean of the values obtained with 11 different KIR2DL1-Fc (2DL1*001, *003, *004, *007, *008, *012, *020, *021, *023 *024, *025). Each individual binding value was normalized to the binding of the W6/32 antibody before calculating the average. (B) Binding of KIR2DL1*022-Fc and KIR2DL2/3-Fc fusion proteins to C1-bearing HLA-B and -C allotypes. For each C1-bearing HLA-B and HLA-C allotype, the KIR2DL2/3 binding is the mean of the values obtained with 16 KIR2DL-Fc fusion proteins (2DL1*022; 2DL2*001,*003, *004, *006, 009 *011; and 2DL3*001, *005, *008, *009, *011, *013, *015, *016, *018). The proteins were tested against microbeads coated with one of nine C1 HLA-C or two C1 HLA-B allotypes. Each individual binding value was normalized to the binding to that of the W6/32 antibody before calculating the average. (C) Variable cell-surface expression of KIR2DL1. FLAG-tagged KIR2DL1 allotypes were transfected into HeLa cells. Cell-surface expression was detected using FLAG-specific antibody and analysis by flow cytometry. MFI = median fluorescence intensity. The experiment was performed in triplicate, error bars give the standard deviation. The difference between 2DL1*012 and 2DL1*026 is statistically significant as assessed by a two-tailed t-test.

The interactions of KIR2DL with HLA-C are not only diversified by KIR2DL1 and KIR2DL2/3 polymorphism, but also by polymorphism within the subsets of C1-bearing and C2-bearing HLA-C allotypes. Binding to C2 by the 11 KIR2DL1 allotypes varied over half an order of magnitude and with a similar hierarchy for each of the KIR allotypes (Fig 2A). Thus HLA-C*15:02 is always the strongest ligand for KIR2DL1 and HLA-C*04:01 the weakest. Analogous patterns were observed for the binding of 2DL1*022 and the 15 KIR2DL2/3 allotypes to C1-bearing HLA-B and -C allotypes (Fig 2B). Here, HLA-B*73:01 is the strongest ligand for 2DL1*022 and KIR2DL2/3 and HLA-C*16:01 the weakest. The basis for these hierarchies within the C1- and C2-bearing allotypes arise from either the differing peptide repertoires presented by specific HLA-C or by polymorphism at sites other than position 80 that defines the C1 and C2 epitopes.

### KhoeSan specific 2DL1*026 lacks a cytoplasmic tail but is cell-surface expressed

KIR2DL1*026 and 2DL1*012 encode identical extracellular domains that bind C2 with high avidity and specificity (Fig 1B). To determine if 2DL1*026, which lacks a cytoplasmic tail, reaches the cell-surface, we examined the expression of FLAG-tagged 2DL1*026 and 2DL1*012 in transiently transfected HeLa cells. For comparison, eight other KIR2DL1 allotypes were included in the analysis (Fig 2C). KIR2DL1*026 is cell-surface expressed at a significantly lower level than 2DL1*012 (p = 0.0087), but within the range observed for other KIR2DL1 allotypes. Although KIR2DL1*026 cannot mediate NK cell inhibition directly, because it lacks a cytoplasmic domain, it could have indirect effects, either by preventing C2 from binding to other receptors or by contributing to the adhesive interactions of NK cells with target cells. That 2DL1*014 is not cell-surface expressed and cannot bind HLA class I suggests that its defining residue, serine 179, prevents proper protein folding. Other KIR allotypes with impaired folding that causes intracellular retention have been described [35–37].

### Characterizing the KhoeSan population is a high frequency of weak C2 receptors

**Fig 3:**
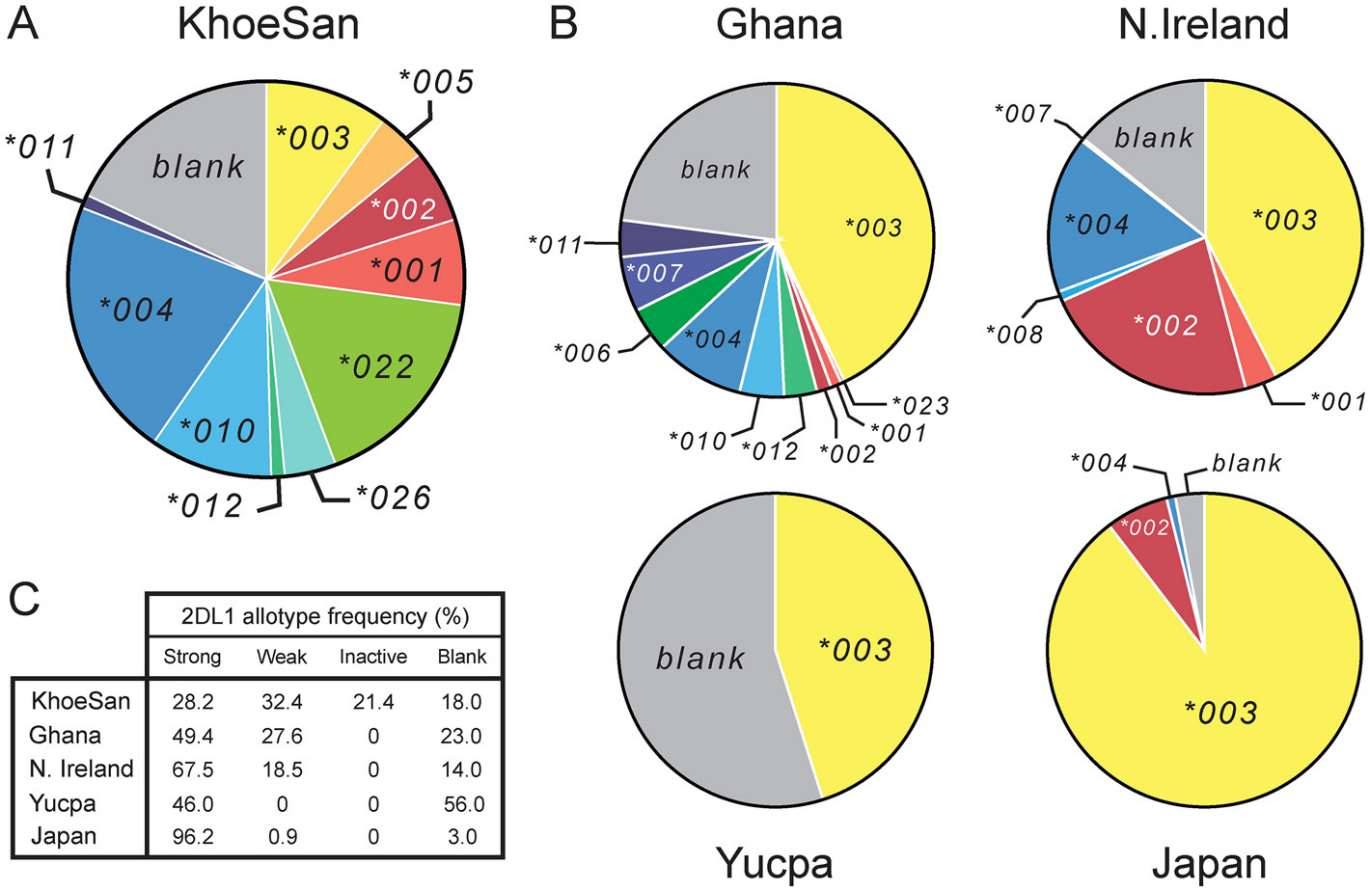
The KhoeSan have high *KIR2DL1* diversity compared to other human populations. (A and B) The pie charts show the number and relative frequencies of *KIR2DL1* alleles in the KhoeSan of Southern Africa (A), and four other populations representing four continents (B): the Ga-Adangbe from Ghana in Western Africa [14], Northern Ireland Caucasians from Europe [21], Japanese from East Asia [24] and Yucpa Amerindians from South America [20]. The 'blank' is the frequency of *KIR* haplotypes that lack the *KIR2DL1* gene. (C) Also compared in the five populations are the frequencies of strong KIR2DL1, weak KIR2DL1, KIR2DL1 that are not inhibitory C2 receptors (inactive) and the absence of KIR2DL1 (blank). The definition and designation of these KIR2DL1 categories are given in S4 Fig.

Unlike some other populations, there is no single *2DL1* allele that is present at high frequency in the KhoeSan (Fig 3 and S1A Fig). The ten KhoeSan *2DL1* alleles vary in frequency from 1.1–21.3%. In addition, 18% of KhoeSan *KIR* haplotypes lack the *KIR2DL1* gene, constituting an eleventh allele: the 'blank'. The frequency of 2DL1*022, (17.2%) is more than double that of 2DL1*001 (7.0%), the parental allele from which it evolved. Likewise, 2DL1*026 (4.2%) has a higher frequency than 2DL1*012 (1.1%), the parental allele from which it evolved. The impact of both 2DL1*022 and 2DL1*026 has been to reduce the capacity of KIR2DL1 to function as an inhibitory C2 receptor in the KhoeSan.

This effect of the KhoeSan-specific *KIR2DL1* alleles is reinforced by the relatively low frequency in the KhoeSan of other alleles encoding strong inhibitory C2 receptors (2DL1*001, *002, *003 and *005) and relatively high frequency of alleles encoding weaker inhibitory C2 receptors. Included in the latter are the ‘blank’, the 2DL1*004, 2DL1*010 and 2DL1*011 receptors that have reduced avidity for C2 (Fig 1B and S4 Fig) and the 2DL1*004 and 2DL1*011 allotypes that have reduced signaling capacity caused by the cysteine residue at position 245 [38] (Fig 1A). In sum, the frequency of weak or inactive 2DL1 allotypes in the KhoeSan is 71.8%, whereas the 28.2% frequency of strong 2DL1 allotypes in the KhoeSan is much lower than that of other populations (Fig 3C).

### KIR2DL1*022 and 2DL1*026 are present on conserved haplotypes that are recently evolved

**Fig 4:**
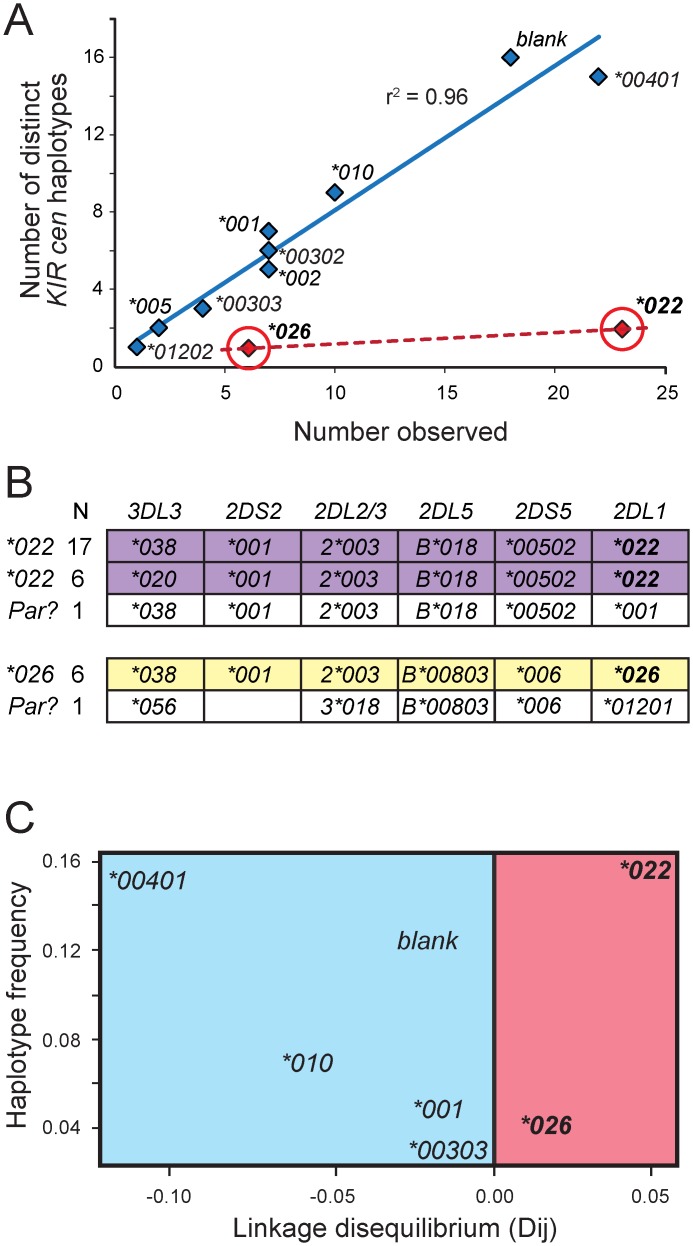
KIR2DL1*022 and 2DL1*026 are of recent origin compared to other KhoeSan *KIR2DL1* alleles. (A) For each KhoeSan *KIR2DL1* allele, the number of centromeric *KIR* haplotypes on which the allele is present in the KhoeSan (number observed) is plotted against the number of different (distinct) haplotypes on which the allele is present. In total, 110 haplotypes were analyzed. Haplotypes that lack *KIR2DL1* are denoted ‘blank’. The r^2^ was calculated from Pearson correlation of the alleles shown in blue. This analysis excluded 2DL1*022 and *026 (shown in red). (B) Shows the allele content of centromeric *KIR* haplotypes containing either 2DL1*022 (purple) or 2DL1*026 (yellow). The observed number of each haplotype is given on the left. Also shown (in white) are the KhoeSan haplotypes that are the putative parents (Par?) of the derived 2DL*022-containing and 2DL1*026-containing haplotypes. The putative parents are the haplotypes that differ from the derived haplotypes by the least number of nucleotide substitutions. (C) Plot of haplotype frequency against linkage disequilibrium (LD). The analysis was conditioned so that 2DL2*003-bearing haplotypes were analyzed. The figure illustrates the high level of linkage disequilibrium observed for haplotypes containing 2DL1*022 and 2DL1*026 suggesting they appeared more recently in the KhoeSan population than other *KIR2DL1* alleles.

To examine the genetic background of KIR2DL1*022 and KIR2DL1*026, we determined structures for the *KIR2DL1*-containing centromeric region of KhoeSan *KIR* haplotypes. Extensive diversity was observed, there being 70 different haplotypes among a total of 110 haplotypes characterized from 55 unrelated individuals. For each *KIR2DL1* allele we determined how many different haplotypes have the allele and what their frequencies are in the KhoeSan. Because the linkage disequilibrium (LD) between nine of the eleven KhoeSan *2DL1* alleles and other genes of the centromeric region is low, there is a strong positive correlation (r^2^ = 0.96) between an allele's frequency and the number of different haplotypes on which it occurs (Fig 4A). For example, a total of seven haplotypes have 2DL1*001 and they are all different in their linked *KIR* alleles and genes (Fig 4A). That we find numerous different haplotypes reflects the high diversity of KhoeSan genomes [10,16]. Dramatic exceptions to this pattern are the haplotypes containing the KhoeSan specific *KIR2DL1* alleles, which are in complete LD with the other centromeric *KIR* genes and alleles. Among the 23 haplotypes containing 2DL1*022 only two are unique, and they differ only in *KIR3DL3* at the centromeric end of the *KIR* locus (Fig 4B). The six haplotypes containing 2DL1*026 are all identical (Fig 4B). The high LD across these haplotypes shows that they have not been broken and mixed by meiotic recombination, which is consistent with their recent evolution [39] (Fig 4C).

## Discussion

Our study shows how *KIR2DL1* polymorphism has given rise to NK cell receptors that vary substantially in their capacity to recognize HLA-C and propagate intracellular signals. Emphasizing the value of defining structural and functional KIR variation at high resolution is our discovery in the KhoeSan of two unusual allotypes of KIR2DL1, the inhibitory NK cell receptor for the C2 epitope of HLA-C. The alleles encoding these allotypes were derived by point mutation from older, more widespread *KIR2DL1* alleles that encode strong, inhibitory C2 receptors. In stark contrast to the parental allotypes, neither progeny is a strong, inhibitory C2 receptor. KIR2DL1*026 has no capacity for signal transduction and 2DL1*022 recognizes C1 with specificity and avidity that exceeds that of any KIR2DL2/3 allotype, the archetypal C1 receptor. The methionine to lysine substitution at position 44 that defines KIR2DL1*022 occurs within the HLA-C binding site of the KIR [5]. Here, residue 44 in the D1 domain of the KIR interacts with residue 80 of the α_1_ domain of HLA-C. For KIR2DL1*001, the parent allele of KIR2DL1*022, methionine 44 binds to lysine 80 of the C2 epitope of HLA-C [5,7]. In contrast, lysine 44 in KIR2DL1*022 binds to asparagine 80 of the C1 epitope of HLA-C.

KIR2DL1*022 is the most vivid example of how genetic polymorphism can change KIR specificity for HLA class I. For other allotypes of KIR2DL1 and KIR2DL2/3, the effects of their defining substitutions can act to alter different functional properties: receptor avidity [31,32,40], stability, cell-surface abundance and signal transduction [38]. Throughout the KIR molecule are sites where natural substitutions affect receptor functions. Many of these are away from the HLA-C binding site and likely involve conformational changes, including ones that affect the relative orientation of the extracellular D1 and D2 domains that combine to form the binding site [31,40]. That KIR2DL1*022 and 2DL1*026 have lost their parents’ capacity to function as inhibitory C2 receptors, exemplifies a more widespread trend in the KhoeSan. That is an accumulation of KIR2DL1 allotypes with low avidity for HLA-C2 or weakened signaling function, as well as *KIR B* haplotypes lacking the *KIR2DL1* gene (Fig 3C).

**Fig 5:**
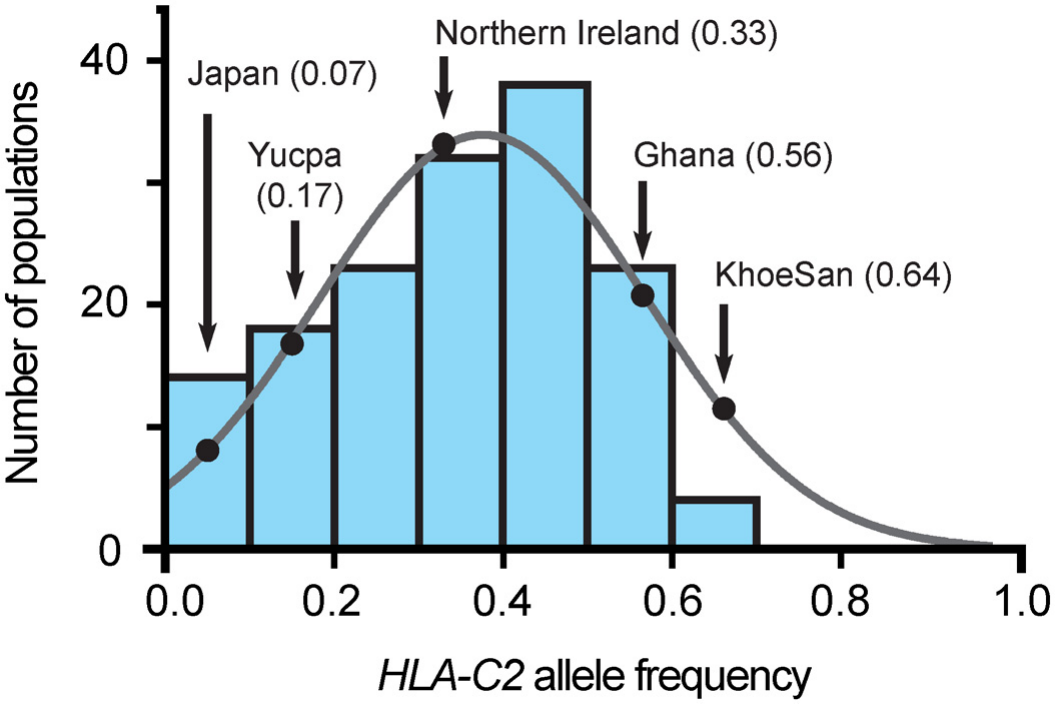
The C2 frequency in the KhoeSan is unusually high. Each of the seven blue-shaded vertical bars gives the number of populations, of 140 considered [34], that have a C2 frequency within the range covered by the bar, given on the horizontal axis. The frequency data are not significantly different from a normal distribution (grey line). The black-shaded dots on the curve give the frequencies for the KhoeSan and the four other populations for which *KIR2DL1* allele frequencies are given in Fig 3.

In human populations worldwide there is an inverse correlation between the frequency of HLA-C allotypes carrying the C2 epitope and the frequency of the *KIR A* haplotypes encoding strong KIR2DL1 allotypes. This correlation reflects the increased risk of spontaneous abortion, preeclampsia, and low birth-weight that is associated with pregnancies in which a *KIR A* homozygous mother who lacks the C2 epitope is carrying a fetus that expresses a C2 epitope of paternal origin [41,42]. In these pregnancies, the interaction of paternal C2 on extravillous trophoblast cells with maternal uterine NK cells expressing the strong KIR2DL1 encoded by *KIR A* haplotypes can lead to incomplete placentation. In general, Africans have a higher frequency of the C2 epitope than other populations and the C2 frequency of the KhoeSan is particularly high (63.4%; Fig 5). The reasons for the high C2 frequency are unknown, but may include protection against specific diseases though interaction of C2-expressing HLA-C with NK cells or CD8 T cells. Thus the emergence of 2DL1*022 and 2DL1*026, as well as the general increase of weaker inhibitory KIR2DL1 allotypes, in the KhoeSan could have acted to reduce the incidence of preeclampsia. In this manner, the KhoeSan retained the ability to both fight infection and reproduce efficiently.

In assessing the effect of a high C2 frequency on the KhoeSan, it is informative to consider the Yucpa, an indigenous South American population that has a low frequency of C2 and a high frequency of C1 (82.7%) [20]. Accompanying the abundance of C1 are two Yucpa-specific *KIR2DL3* alleles, both arising by point mutation of the older, widespread 2DL3*001. KIR2DL3*009 has lower C1 avidity than 2DL3*001 and 2DL3*008N is non-functional. These Yucpa specific 2DL3 have a frequency of 41.8% compared to 8.2% for their 2DL3*001 parent. In the Yucpa, the high C1 frequency combines with a much-reduced frequency of strong inhibitory C1 receptors, whereas in KhoeSan, the high C2 frequency combines with a much-reduced frequency of strong inhibitory C2 receptors. These analogous behaviors at the two extremes of the frequency spectrum appear to reflect a buffering mechanism that maintains a balance between C1, C2 and their inhibitory receptors in human populations.

One possibility is that 2DL1*022 and 2DL1*026 increased in frequency as a consequence of genetic drift. Thus, they would represent two of the many private alleles that are present in the KhoeSan because of their unique demographic history [12]. Unlike other African hunter-gatherer groups, the KhoeSan have maintained a large effective population size and high levels of genetic diversity [10,43]. These characteristics argue against the KhoeSan having been subject to a classic bottleneck of the type experienced by other African hunter-gatherer populations, such as the Tanzanian Hadza [10], or migrant modern humans who left Africa and populated other continents [44]. An alternative interpretation is that 2DL1*022 and 2DL1*026 rose in frequency in the KhoeSan under positive selection. Supporting this model are the distinctive functional properties of the 2DL1*022 and 2DL1*026 proteins, the evidence for balancing selection at the *KIR* locus [20,24,45,46] and the evidence for diversifying selection at position 44, where lysine determines the unique functionality of KIR2DL1*022 [9]. To establish if drift or selection is responsible for emergence of the new, variant KIR, will require extensive demographic simulations and the development of appropriate programs that simulate co-evolution between unlinked, highly polymorphic loci.

## Materials and Methods

### Ethics statement

Sampling of the ≠Khomani San in Upington, South Africa and neighboring villages occurred in 2006. Institution Review Board (IRB) approval was obtained from Stanford University [Protocol 13829] for assessment of genetic diversity and ancestry inference. Individuals who were still living in 2011 were re-consented (IRB approved from Stanford University and Stellenbosch University, South Africa). ≠Khomani N|u-speaking individuals, local community leaders, traditional leaders, non-profit organizations and a legal counselor were all consulted regarding the aims of this research, prior to collection of DNA. All individuals consented orally to participation, with a second, local native speaker witnessing and were re-consented with written consent. DNA was collected via saliva and all individuals were as described in previous studies [10,26].

### Study populations

Genomic DNA samples were isolated from saliva samples donated by 61 KhoeSan individuals of the ≠Khomani San population as described by Henn et al. [10]. *KIR2DL1*, *KIR2DL2/3* and *HLA-C* allele frequencies were determined for 55 unrelated individuals. The additional six individuals comprised five additional family members of two of the 55 unrelated individuals, and a sibling of another. The sequences and frequencies of KhoeSan *KIR* and *HLA-C* alleles were compared to those of Ghanaians [14], Northern Irish [21] Japanese [24] and South Amerindians [20], and also to three non-KhoeSan hunter-gatherer populations. These comprised 20 Mbuti and 20 Baka Pygmies from The Democratic Republic of Congo and Cameroon, and 52 Hadza from northern Tanzania [10]. We also analyzed the *KIR* sequence data of 100 Zulus from South Africa [27]. Allele frequencies for the C1 and C2 epitopes of HLA-C were determined using data deposited at www.allelefrequencies.net [34]. The 140 populations analyzed were chosen for being anthropologically well characterized, for having minimal admixture with other populations, and for having a size of 40 individuals or more. This panel of populations is described in Abi Rached et al. [33].

### High-resolution *KIR2DL1* and *KIR2DL2/3* genotyping

Nucleotide sequences were determined for all exons of *KIR2DL1 and KIR2DL2/3* genes from sixteen randomly selected unrelated KhoeSan individuals as well as the seven-member family. Sequences for two previously unknown alleles, KIR2DL1*022 (GU323355) and KIR2DL1*026 (JX523630) were confirmed by re-amplification, cloning and sequencing, as described [26]. A pyrosequencing-based method for allele-level *KIR2DL1* and *KIR2DL2/3* genotyping [14], was expanded to include detection of the new KhoeSan variants (S5 Fig). This method provides a semi-quantitative measure of SNP genotypes (the peak-height ratio) that determines both allele identity and copy-number genotype [14]. Centromeric *KIR* haplotypes were characterized as described [14], with modification to accommodate the newly-discovered 3DL3*038 and 2DL5B*018 alleles [26]. Pyrosequencing and standard Sanger sequencing were used to determine the *2DL1* alleles present in the Pygmy and Hadza populations.

### High-resolution *HLA* genotyping

The 61 KhoeSan individuals were *HLA-C* genotyped at allele-level resolution using bead-based SSOP hybridization (One Lambda) and detection by a Luminex-100 instrument (Luminex corp. Austin, TX).

### KIR nomenclature

*KIR* genes and alleles were named by the KIR nomenclature committee [47] formed from members of the WHO Nomenclature Committee for factors of the HLA system, and the HUGO Genome Nomenclature Committee. A curated database is available at http://www.ebi.ac.uk/ipd/kir/ [47].

### Searching for KIR2DL1*022 and KIR2DL1*026 alleles in the 1000 Genomes and African Genome Variation Project datasets

The high-coverage exome data from the May 2013 release of the 1000 Genomes project [25] were used to determine the frequency of KIR2DL1*022 and KIR2DL1*026 in populations worldwide. All read-pairs that map to the *KIR* regions (Build Hg19: chr19:55,228,188–55,383,188 and GL000209.1) were extracted using SAMtools 0.1.18 [48]. For 39 individuals the data have insufficient coverage and were excluded from the analysis, which was performed on data from 2,496 individuals representing 26 populations (S2B Fig). Individual fastq files were probed using locus-specific and allele-specific sequence-string searches. Where required, individual fastq files were filtered for locus-specificity using Bowtie (version 0.12.7) [49], aligned to references and the SNP genotypes inspected manually. As controls we included data from eight KhoeSan individuals who had previously been sequenced using Illumina whole-exome paired end technology [26] and, independently, KIR genotyped to allele-level resolution by pyrosequencing [14]. Three of the eight individuals have KIR2DL1*022, and one other has KIR2DL1*026.

We used the same method to determine the frequencies of 2DL1*022 and 2DL1*026 in 100 Zulus whose genomes were sequenced as part of the African Genome Variation project [27]. Zulus are a Bantu-speaking population from southern Africa, who show evidence for recent admixture with the KhoeSan [11,18]. For each Zulu individual having either 2DL1*022 or 2DL1*026 we used manual inspection of sequence reads mapped to each *KIR* gene to infer the likely centromeric *KIR* haplotype structure.

### Binding assay of KIR-Fc fusion proteins to beads coated with HLA class I

KIR-Fc fusion proteins were generated from insect cells (cabbage looper moth *Hi5* cells, kindly provided by Prof. K.C. Garcia, Stanford University) infected with baculovirus as described [50]. The KIR-Fc fusion protein corresponding to each 2DL1, 2DL2 and 2DL3 allotype was tested for binding to a panel of microbeads, each of which is coated with one of 31 HLA-A, 50 HLA-B and 16 HLA-C allotypes (LabScreen Single-Antigen Beads, One Lambda, lot #8). To account for differences in the amount of HLA class I protein coating each bead, the binding of each KIR-Fc fusion protein was normalized to the binding of W6/32, a monoclonal antibody detecting a common epitope of HLA class I. Binding values were calculated using the formula (specific binding—bead background fluorescence)/(W6/32 binding—bead background fluorescence).

### Cell-surface expression of KIR2DL1 in transiently transfected HeLa

Recombinant cDNA encoding the extracellular, stem, transmembrane and cytoplasmic domain (amino acids 1–336) of KIR2DL1*003 with an N-terminal 3X FLAG-tag was manufactured by Genscript (Piscataway, NJ) and cloned into the pcDNA3.1+ expression vector. Site-directed mutagenesis was performed with the QuikChange Kit (Stratagene), according to the manufacturer’s instructions, to generate nine further *KIR2DL1* variants. HeLa cells (ATCC Cell Lines, VA) were plated in 15.6mm wells at 5 x 10^4^ cells/well in 500μl DMEMc for 24hrs and then transfected with a pcDNA3.1+ vector encoding FLAG-tagged KIR2DL1 allotypes using the Fugene transfection reagent (Promega). After 36h, adherent cells were dissociated from the wells using 200μl 0.05% trypsin EDTA solution and stained with 25μl mouse polyclonal FITC-conjugated FLAG-specific antibody (Sigma-Aldrich) at a final concentration of 3μg/ml. Cells expressing FLAG-tagged KIR2DL1 allotypes were detected by flow cytometry (Accuri C6 cytometer, BD Biosciences). Dead cells were identified by staining with propidium iodide and excluded from the analysis. The median fluorescence intensity (MFI) of FITC-conjugated anti-FLAG antibody bound to each positively staining cell was used as a measure of the cell-surface expression of KIR2DL1. At least 50,000 such cells were analyzed in each experiment. Three independent transfections were performed for each allotype.

### Population and molecular genetic analysis

The *KIR* locus has extensive structural and allelic polymorphism [8,51], as well as recombination hotspots that flank the centromeric *KIR* region [52,53]. These characteristics preclude the use of methods that use SNP analysis and the identification of regions of extended haplotype homozygosity as evidence for selection [54–56]. We examined the patterns of LD associated with specific alleles, using a method designed for analysis of a polymorphic multigene family [39,57]. This approach was applied to the analysis of the haplotypes in centromeric region of the KhoeSan *KIR* locus, the regions containing the *KIR2DL1* gene.

## Supporting Information

S1 Fig Comparison of the *KIR2DL1*, *KIR2DL2/3* and *HLA-C* allele frequencies in five human populations. The *KIR2DL1* (A) and *KIR2DL2/3* (B) alleles of the KhoeSan are compared to those of the Ga-Adangbe, a Ghanaian population, the Caucasian population of Northern Ireland, the Yucpa South Amerindians from Venezuela and Japanese [14,20,21,24]. (C) Shown are the frequencies of *HLA-C* alleles from each of the five populations described above. The alleles are grouped into those encoding HLA-C allotypes with the C1 epitope and those encoding HLA-C allotypes with the C2 epitope.
(PDF)

S2 Fig KIR2DL1*022 and 2DL1*026 evolved in the KhoeSan after their divergence from other modern human populations. (A) Shown are the results from a search, of whole-exome genome data from eight KhoeSan individuals [26], for sequence reads corresponding to *KIR2DL1* and to the 2DL1*022 and 2DL1*026 alleles. Shown on the left are the *KIR2DL1* genotypes of each individual as assessed by pyrosequencing. Shown on the right are the numbers of *KIR2DL1*, 2DL1*022 and 2DL1*026 reads obtained from the exome data and the percentage of total *2DL1*-specific reads that they constitute. (B) Shown is the number of individuals from each of 26 populations covered by the 1000 Genomes dataset [25]. (C) Shown is the number of individuals from the 1000 Genomes project [25] and African Genome Variation project (AGVP) [27] datasets that tested positive for 2DL1*022 and 2DL1*026, using the same probes as for panel (A). For no individual in the 1000 Genomes dataset did >1% of the reads that covered the SNP location match the tested allele (2DL1*022 or 2DL1*026), whereas for the control individuals the read ratio matched the expected genotype. Five Bantu-speaking Zulu individuals from the AGVP dataset had reads consistent with heterozygosity for either 2DL1*022 or 2DL1*026. Also shown are the frequencies of *3DL3*, *2DL1*, 2DL1*022 and 2DL1*026 in two non-KhoeSan hunter-gatherer populations: the Mbuti and Baka Pygmies (central Africa) and the Hadza (Tanzania). Genotype data for these populations were obtained by whole-exome sequencing.
(PDF)

S3 Fig In the Bantu-speaking Zulu population of southern Africa, 2DL1*022 and 2DL1*026 are present on that same *KIR* haplotypes as in the KhoeSan. (A) Allele content of centromeric *KIR* haplotypes containing either 2DL1*022 (purple) or 2DL1*026 (yellow) that were defined from analysis of 61 KhoeSan individuals. The number of haplotypes observed is given on the left under 'N'. Also shown are KhoeSan *KIR* haplotypes that are putative parents (Par?) of the haplotypes containing 2DL1*022 or 2DL1*026 haplotypes (white boxes). (B) Inferred allele content of centromeric *KIR* haplotypes containing either 2DL1*022 (purple) or 2DL1*026 (yellow) that were defined from analysis of 100 Bantu-speaking Zulu individuals. The number of haplotypes observed is given on the left under 'N'.
(PDF)

S4 Fig KIR2DL1 allotypes can be classified into functional groups based on HLA-C2 binding and signaling capacity. (A) KIR2DL1 allotypes were classified into three functional groups on the basis of their binding to C2 targets and their predicted signaling capacity. KIR2DL1 allotypes were classified as strong if they had a mean binding to C2 that is greater than 50% of the strongest known 2DL1 allotype (2DL1*020). Allotypes were considered weak if they had either a mean binding to C2 that is less than 50% of the strongest 2DL1 allotype or if they have a cysteine residue at position 245, which is known to reduce inhibitory signaling capacity [38]. KIR2DL1 allotypes with no detectable binding to HLA-C2 or no capacity to transduce an inhibitory signal were classified as inactive. (B) Table showing the classification of KIR2DL1 allotypes into groups of strong, weak and inactive receptors.
(PDF)

S5 Fig Oligonucleotide primers used to amplify single *KIR2DL1* exons. An initial PCR was performed using the amplification primers shown in the upper panel. Pyrosequencing was performed following a second (nested) amplification; (o-) indicates biotin and (nnnn-) indicates random oligonucleotides (to prevent fragment looping). The pyrosequencing reactions were performed using the primers shown in the lower panel. When required, standard Sanger sequencing was performed using the amplification primers.
(PDF)
