# Supplementary figures and images for: Loss and Gain of Natural Killer Cell Receptor Function in an African Hunter-Gatherer Population
Source: PLoS Genet. 2015 Aug 20;11(8):e1005439. doi: 10.1371/journal.pgen.1005439 (PMC4546388; doi:10.1371/journal.pgen.1005439)

Figure S1

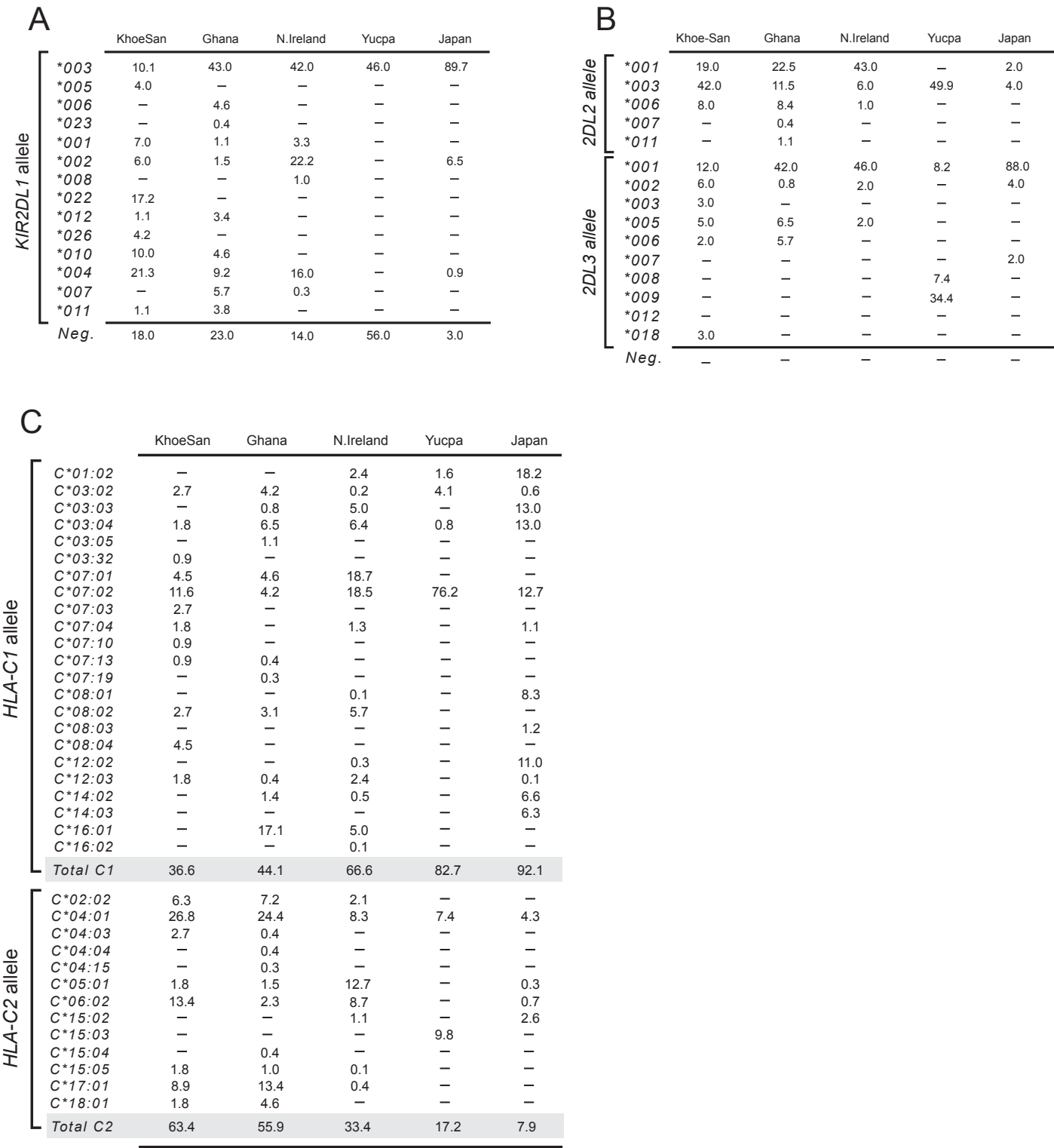

Supplement: S1 Fig — The KIR2DL1 (A) and KIR2DL2/3 (B) alleles of the KhoeSan are compared to those of the Ga-Adangbe, a Ghanaian population, the Caucasian population of Northern Ireland, the Yucpa South Amerindians from Venezuela and Japanese [14,20,21,24]. (C) Shown are the frequencies of HLA-C alleles from each of the five populations described above. The alleles are grouped into those encoding HLA-C allotypes with the C1 epitope and those encoding HLA-C allotypes with the C2 epitope. (PDF) [file pgen.1005439.s001.pdf]
