# Supplementary material for: Loss and Gain of Natural Killer Cell Receptor Function in an African Hunter-Gatherer Population
Source: PLoS Genet. 2015 Aug 20;11(8):e1005439. doi: 10.1371/journal.pgen.1005439 (PMC4546388; doi:10.1371/journal.pgen.1005439)
Supplement: S2 Fig — (A) Shown are the results from a search, of whole-exome genome data from eight KhoeSan individuals [26], for sequence reads corresponding to KIR2DL1 and to the 2DL1*022 and 2DL1*026 alleles. Shown on the left are the KIR2DL1 genotypes of each individual as assessed by pyrosequencing. Shown on the right are the numbers of KIR2DL1, 2DL1*022 and 2DL1*026 reads obtained from the exome data and the percentage of total 2DL1-specific reads that they constitute. (B) Shown is the number of individuals from each of 26 populations covered by the 1000 Genomes dataset [25]. (C) Shown is the number of individuals from the 1000 Genomes project [25] and African Genome Variation project (AGVP) [27] datasets that tested positive for 2DL1*022 and 2DL1*026, using the same probes as for panel (A). For no individual in the 1000 Genomes dataset did >1% of the reads that covered the SNP location match the tested allele (2DL1*022 or 2DL1*026), whereas for the control individuals the read ratio matched the expected genotype. Five Bantu-speaking Zulu individuals from the AGVP dataset had reads consistent with heterozygosity for either 2DL1*022 or 2DL1*026. Also shown are the frequencies of 3DL3, 2DL1, 2DL1*022 and 2DL1*026 in two non-KhoeSan hunter-gatherer populations: the Mbuti and Baka Pygmies (central Africa) and the Hadza (Tanzania). Genotype data for these populations were obtained by whole-exome sequencing. (PDF) [file pgen.1005439.s002.pdf]

Figure S2

A

ID	2DL1 genotype		Number of specific reads: *2DL1*	*2DL1*022*	*2DL1*026*	% 2DL1
SA006	**00401*	**00401*	51	0	0	0
SA008	**01202*	X	186	0	0	0
SA011	**00401*	**00401*	112	0	0	0
SA012	**00401*	**00401*	91	0	0	0
SA035	**022*	X	22	22	0	100
SA051	**00302*	**022*	159	77	0	48.4
SA052	**026*	X	36	0	36	100
SA054	**00401*	**022*	152	72	0	47.4

B

Population	Source	Count
Chinese Dai	Xisghuangbanna, China	98
Han Chinese	Beijing, China	101
Japanese	Tokyo, Japan	99
Kinh	Ho Chi Minh City, Vietnam	101
Southern Han Chinese	China	107
Total East Asian Ancestry		506
Bengali	Bangladesh	86
Gujurati Indian	Houston, Texas	105
Indian Telegu	United Kingdom	103
Punjabi	Lahore, Pakistan	96
Sri Lankan Tamil	United Kingdom	103
Total South Asian Ancestry		493
African Ancestry	Southwestern USA	66
African Caribbean	Barbados	95
Esan	Nigeria	99
Gambian	Western Division, The Gambia	113
Luhya	Webuye, Kenya	98
Mende	Sierra Leone	85
Yoruba	Ibadan, Nigeria	99
Total African Ancestry		655
British	United Kingdom	89
Finnish	Finland	98
Iberian	Spain	106
Toscani	Italy	108
Utah European Ancestry	Utah, USA	96
Total European Ancestry		497
Columbian	Medellin, Columbia	93
Mexican Ancestry	Los Angeles, California	63
Peruvian	Lima, Peru	84
Puerto Rican	Puerto Rico	105
Total Americas Ancestry		345
Total		2496

C

KIR	Positive individuals: 1000 Genome	AGVP Zulu	Mbuti Pygmy	Baka Pygmy	Hadza
3DL3	2496	100	20	20	52
2DL1	2459	97	19	20	52
*2DL1*022*	0	3	0	0	0
*2DL1*026*	0	2	0	0	0
