# Supplementary material for: Loss and Gain of Natural Killer Cell Receptor Function in an African Hunter-Gatherer Population
Source: PLoS Genet. 2015 Aug 20;11(8):e1005439. doi: 10.1371/journal.pgen.1005439 (PMC4546388; doi:10.1371/journal.pgen.1005439)
Supplement: S3 Fig — (A) Allele content of centromeric KIR haplotypes containing either 2DL1*022 (purple) or 2DL1*026 (yellow) that were defined from analysis of 61 KhoeSan individuals. The number of haplotypes observed is given on the left under 'N'. Also shown are KhoeSan KIR haplotypes that are putative parents (Par?) of the haplotypes containing 2DL1*022 or 2DL1*026 haplotypes (white boxes). (B) Inferred allele content of centromeric KIR haplotypes containing either 2DL1*022 (purple) or 2DL1*026 (yellow) that were defined from analysis of 100 Bantu-speaking Zulu individuals. The number of haplotypes observed is given on the left under 'N'. (PDF) [file pgen.1005439.s003.pdf]

Figure S3

A

		N	*3DL3*	*2DS2*	*2DL2/3*	*2DL5*	*2DS5*	*2DL1*
KhoeSan		17	**038*	**001*	*2*003*	*B*018*	**00502*	****022***
		6	**020*	**001*	*2*003*	*B*018*	**00502*	****022***
	Par?	1	**038*	**001*	*2*003*	*B*018*	**00502*	**001*
		6	**038*	**001*	*2*003*	*B*00803*	**006*	****026***
	Par?	1	**056*		*3*018*	*B*00803*	**006*	**01201*

B

	N	*3DL3*	*2DS2*	*2DL2/3*	*2DL5*	*2DS5*	*2DL1*
Zulu	3	**038*	**001*	*2*003*	*B*018*	**00502*	****022***
	1	**038*	**001*	*2*003*	*B*00803*	**006*	****026***
	1	**056*	**001*	*3*018*	*B*00803*	**006*	****026***
