# Supplementary material for: Loss and Gain of Natural Killer Cell Receptor Function in an African Hunter-Gatherer Population
Source: PLoS Genet. 2015 Aug 20;11(8):e1005439. doi: 10.1371/journal.pgen.1005439 (PMC4546388; doi:10.1371/journal.pgen.1005439)
Supplement: S4 Fig — (A) KIR2DL1 allotypes were classified into three functional groups on the basis of their binding to C2 targets and their predicted signaling capacity. KIR2DL1 allotypes were classified as strong if they had a mean binding to C2 that is greater than 50% of the strongest known 2DL1 allotype (2DL1*020). Allotypes were considered weak if they had either a mean binding to C2 that is less than 50% of the strongest 2DL1 allotype or if they have a cysteine residue at position 245, which is known to reduce inhibitory signaling capacity [38]. KIR2DL1 allotypes with no detectable binding to HLA-C2 or no capacity to transduce an inhibitory signal were classified as inactive. (B) Table showing the classification of KIR2DL1 allotypes into groups of strong, weak and inactive receptors. (PDF) [file pgen.1005439.s004.pdf]

Figure S4

A

Signal capacity	Binding capacity for HLA-C2: >50%	<50%	No binding
R245	Strong	Weak	Inactive
C245	Weak	Weak	Inactive
No Signal	Inactive	Inactive	Inactive

B

	Functional classification: Strong	Weak	Inactive
2DL1 allotype	*001 *016<br>*002 *017<br>*003 *018<br>*005 *019<br>*009 *020<br>*012 *023<br>*015	*004 *011<br>*006 *021<br>*007 *024<br>*008 *025<br>*010	*013N<br>*014<br>*022<br>*026
