# Supplementary material for: Loss and Gain of Natural Killer Cell Receptor Function in an African Hunter-Gatherer Population
Source: PLoS Genet. 2015 Aug 20;11(8):e1005439. doi: 10.1371/journal.pgen.1005439 (PMC4546388; doi:10.1371/journal.pgen.1005439)
Supplement: S5 Fig — An initial PCR was performed using the amplification primers shown in the upper panel. Pyrosequencing was performed following a second (nested) amplification; (o-) indicates biotin and (nnnn-) indicates random oligonucleotides (to prevent fragment looping). The pyrosequencing reactions were performed using the primers shown in the lower panel. When required, standard Sanger sequencing was performed using the amplification primers. (PDF) [file pgen.1005439.s005.pdf]

Figure S5

KIR2DL1 and KIR2DS1

	Exon/cDNA position	Forward primer	Reverse primer
Amplification	Exon 1	ACACTAGGTGTCAAATTCTAGC	GCGCTCCCTCCCTCTATTG
		nnnnATAACATCCTGTGCGCTGCT	o-TTGCTTTCCAGGACTCACCA
	Exon 4	ACAGAGGGACAGAGAAGAGT	TGTTTCTCTGTCTCTGTTGGC
		nnnnGACACCAGGAAGGGGAAGC	o-ACTCTGCATCCCAATGACAAT
	Exon 5	ACATGAAGAGCGATGGGGTA	CCTGGAGCCCTGGCCGT
		nnnnCCTCTTCTCCTTCCAGGTCTA	o-GCAGTGGGTCACTTGACTTTG
	Exon 6	CTCAAAGATTTCCACTGAGTG	AGGGTTGCTTCATGACCTAC
		GGGTGCTTGTCCTAAAGAGG	TGTTGTCTCCCTAGAAGACG
	Exons 7-9	nnnnTAACCCCCGACACCTGCAC	o-CCATCCTGCTTCCCCACAT
		o-ATTTCCCTCTCTCCAGGACTCTGA	nnnnTGGGCAGGAGACAACTTTGG
Pyrosequencing	13K	GCACCATGTCGCTCT	
	107R, 110S	CCTCCTGGCCCACCC	
	144R	GAAATCAGAAGAGACAGTCA	
	194W	CTTCTGCACAGAGAGG	
	272V	CTCCATCAGTCGCAT	
	331K, 358R	GTTACTCACTCCCCCTAT	
	404Y	TTCTCTCTCAGCCCAG	
	549Y, 550R, 576Y	CAACGGAACATTCCAG	
	598R, 608R, 621Y	GGGACCTACAGATGCTT	
	796Y, 813R	CATCCTCCTCTTCTTTCT	
	888M, 903R, 907R, 908Y	AAACGCAGTGATTCAAC	
